# Integrated Network Toxicology and Metabolomics Reveal the Ovarian Toxicity Mechanisms of Chronic Carbofuran Exposure in Female Mice

**DOI:** 10.3390/ijms27010090

**Published:** 2025-12-21

**Authors:** Di Liang, Hongyu Su, Xian Ju

**Affiliations:** 1 School of Forensic Medicine, Shanxi Medical University, Jinzhong 030600, China; 2 West China School of Basic Medical Sciences and Forensic Medicine, Sichuan University, Chengdu 610000, China

**Keywords:** carbofuran, ovarian toxicity, network toxicology, metabolomics, reproductive toxicity

## Abstract

Carbofuran, a widely used carbamate pesticide, is an endocrine disruptor with documented reproductive toxicity, yet the mechanisms underlying its ovarian toxicity remain incompletely understood. This study employed integrated network toxicology and untargeted metabolomics to investigate these mechanisms in female C57BL/6J mice that had been chronically exposed to carbofuran (0.5 or 2.0 mg/kg for 45 days, once daily). Methods included histopathological evaluation, serum hormone ELISA, network prediction of toxicity targets, molecular docking, and metabolomics profiling. Results demonstrated that carbofuran exposure induced dose-dependent ovarian damage, including reduced follicular reserve, increased atresia, abnormal corpus luteum, and disrupted hormone levels. Network toxicology identified 38 common targets, with EGFR, GSK3B, APP, and JAK2 as core proteins, indicating potential high affinity. Metabolomics suggests significant alterations in pathways such as phenylalanine, tyrosine, tryptophan biosynthesis and arginine/proline metabolism. Our collective evidence indicates that carbofuran may induce ovarian toxicity through multifaceted mechanisms involving endocrine disruption, oxidative stress, inflammatory activation, and metabolic disturbance. This study provides novel experimental insights into the reproductive toxicity mechanisms of carbofuran, offering a theoretical basis for health risk assessment and intervention strategies.

## 1. Introduction

Exogenous chemicals can act as endocrine disruptors, interfering with the homeostasis of normal hormones in organisms and adversely affecting reproductive health [1]. Carbamate compounds are among these endocrine disruptors. Recent reports indicate that carbamate compounds, such as carbofuran, are associated with endocrine disruption and exhibit reproductive toxicity [2].

Carbamate insecticides are widely used globally for pest control in forestry, horticulture, and agriculture [3]. Carbofuran, with its excellent insecticidal efficacy, is one of the commonly used carbamate pesticides, particularly in developing countries [4]. Due to its water solubility, carbofuran is highly mobile in soil, possesses a long half-life (50 days), and can severely contaminate soil and water resources [5]. Residues of carbofuran enter the food chain through biomagnification, and previous reports have documented harmful effects of carbamate pesticides on non-target organisms [6]. Furthermore, carbofuran poisoning incidents continue to occur worldwide, with numerous cases reported over the past few decades [7,8,9,10].

Carbofuran is an anticholinesterase compound that inhibits acetylcholinesterase activity, leading to the accumulation of acetylcholine at nerve synapses and neuromuscular junctions, resulting in cholinergic symptoms and toxicity to mammals, birds, fish, and wildlife [11]. The inhibition of acetylcholinesterase by carbofuran is unstable, short-lasting, and reversible, allowing spontaneous and rapid reactivation compared to the inhibition induced by organophosphorus compounds [12]. Moreover, some reports show that carbofuran is also associated with endocrine disruption, reproductive disorders, cytotoxicity, and genotoxicity [13]. A growing body of research demonstrates that environmental exposure to carbofuran negatively impacts the reproductive function of various organisms [2,14,15]. Animal studies indicate that carbofuran can cause disruption of the estrous cycle, reduction in the number of healthy follicles, and inhibition of ovarian development [16,17,18]. Although current knowledge exists regarding carbofuran’s toxicity, the potential mechanisms underlying carbofuran-induced ovarian toxicity still require further investigation.

Network toxicology is a novel toxicological research method that can provide new directions for studying compound toxicity mechanisms by integrating and analyzing data from multiple databases [19,20]. Metabolomics, as a prominent technique in toxicology research, detects metabolic changes in organisms to reveal the mechanisms of tissue damage and related adverse processes, thereby complementing the predictive results of network toxicology [21]. The combined use of these two techniques covers more interconnected toxicity mechanisms, enabling a comprehensive interpretation of the biological damage caused by chemicals. Currently, metabolomics studies on carbofuran primarily focus on ecosystem degradation and damage from environmental exposure [22,23,24]; its potential mechanisms in ovarian toxicity have not yet been reported.

To address the aforementioned issues, this study combined network toxicology and untargeted metabolomics approaches to investigate the pathways associated with ovarian toxicity in female mice following chronic carbofuran exposure. By integrating metabolite, target, and pathway information, we systematically elucidated its ovarian toxicity mechanisms, providing an experimental basis for future development of ovarian protection strategies, safeguarding reproductive health, and laying the foundation for research on diseases related to carbofuran exposure.

## 2. Results

### 2.1. Effects on Mouse Ovaries

**Figure 1:**
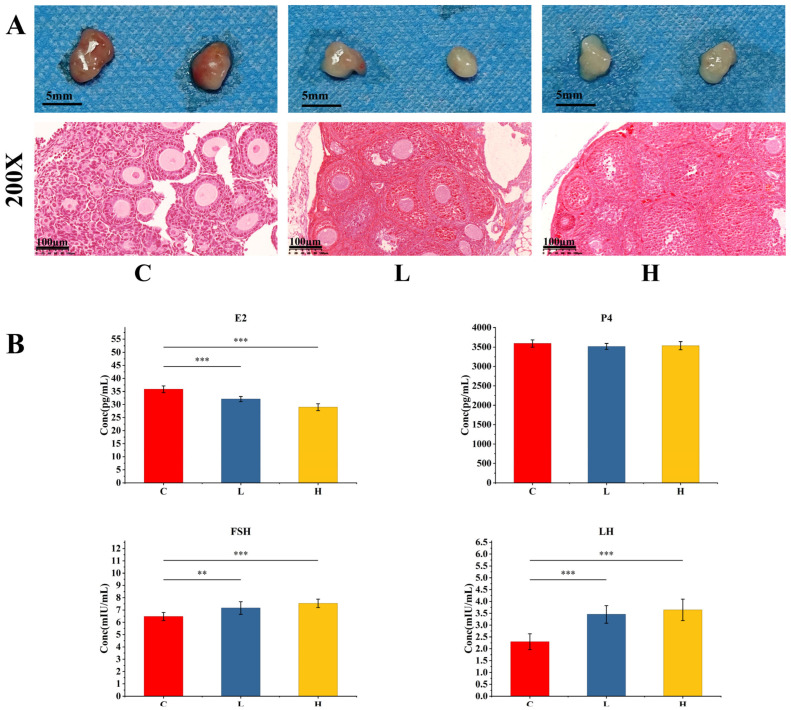
Gross appearance, histopathological images of mouse ovarian tissue, and ELISA results. (**A**) Representative gross appearance (top row) and corresponding histopathological images (bottom row, 200× magnification) of ovarian tissues from the Control, Low-dose, and High-dose groups (left to right). (**B**) ELISA results showing serum levels of E2, P4, FSH, and LH in mouse. Asterisks denote statistical significance: ** for *p* ≤ 0.01 and *** for *p* ≤ 0.001.

Chronic carbofuran exposure caused significant dose-dependent pathological damage to mouse ovaries. Macroscopically, compared with the control group, the ovarian volume was significantly reduced in both the low-dose and high-dose groups (Figure 1). HE-stained sections of ovarian tissue showed a significant decrease in the proportion of primordial follicles in the ovaries of mice in all exposure groups (Figure 1). Concurrently, the numbers of primary and secondary follicles decreased compared to the control group, while the proportion of atretic follicles increased accordingly. Furthermore, the number of corpora lutea increased in the exposed groups, and their morphology was abnormal. In terms of tissue structure, the low-dose group exhibited local disorganization of the ovarian structure, while the high-dose group further showed minor hemorrhaging in the interstitial space and more extensive structural damage. ELISA results showed that compared with the control group, serum estradiol (E2) levels were significantly decreased in the exposure groups, while progesterone (P4) levels showed no significant difference; levels of follicle-stimulating hormone (FSH) and luteinizing hormone (LH) were significantly increased (Figure 1).

### 2.2. Network Toxicology Results

**Figure 2:**
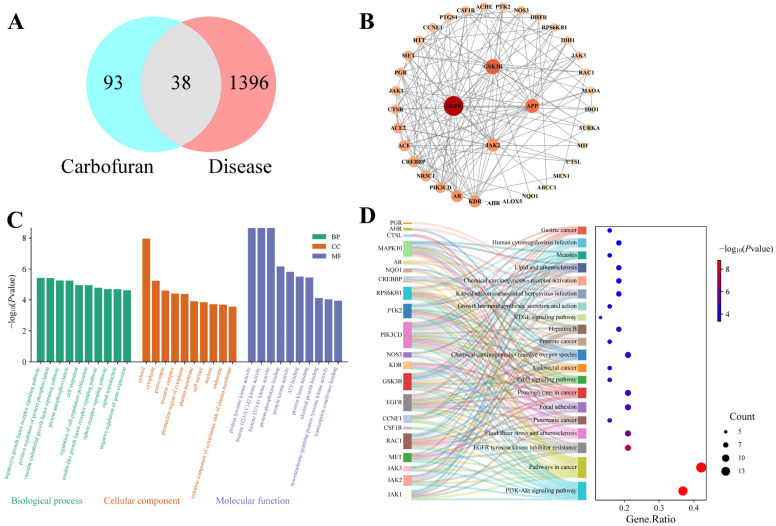
Network toxicology analysis. (**A**) Venn diagram of the overlapping targets between the compound and the disease. (**B**) PPI network of the common targets. (**C**) Gene Ontology (GO) enrichment analysis results, showing the top 10 terms for Biological Process (BP), Cellular Component (CC), and Molecular Function (MF) (from left to right). (**D**) Bubble chart of the top 20 enriched Kyoto Encyclopedia of Genes and Genomes (KEGG) pathways.

Network toxicology was used to explore the potential mechanisms of ovarian toxicity induced by chronic carbofuran exposure in female mice. A total of 131 compound-related targets and 1434 disease-related targets were retrieved, with 38 overlapping targets used to construct a STRING-derived protein–protein interaction (PPI) network (Figure 2). The top 4 nodes ranked by degree value included EGFR, GSK3B, APP, and JAK2.

To further elucidate the biological functions of these common targets, we performed GO and KEGG enrichment analyses. GO analysis returned 490 items, comprising 168 biological process (BP), 39 cellular component (CC), and 89 molecular function (MF) terms. The top 10 entries for each category are visualized in Figure 2. BP terms were primarily enriched in pathways such as hepatocyte growth factor receptor signaling pathway, positive regulation of protein phosphorylation, vascular endothelial growth factor signaling pathway, and protein autophosphorylation. CC terms showed enrichment mainly in cytosol, cytoplasm, postsynapse, and receptor complex. MF terms were principally associated with protein tyrosine kinase activity, histone H2AX Y142 kinase activity, histone H3 Y41 kinase activity, and protein phosphatase binding. KEGG pathway analysis identified 94 signaling pathways, among which the most significant included the PI3K-Akt signaling pathway, pathways in cancer, EGFR tyrosine kinase inhibitor resistance, and fluid shear stress and atherosclerosis. The top 20 pathways are visualized in Figure 2. These pathways broadly involve cell proliferation, survival, inflammatory response, and endocrine regulation, suggesting a potential multi-mechanistic network underlying carbofuran-induced ovarian toxicity.

**Figure 3:**
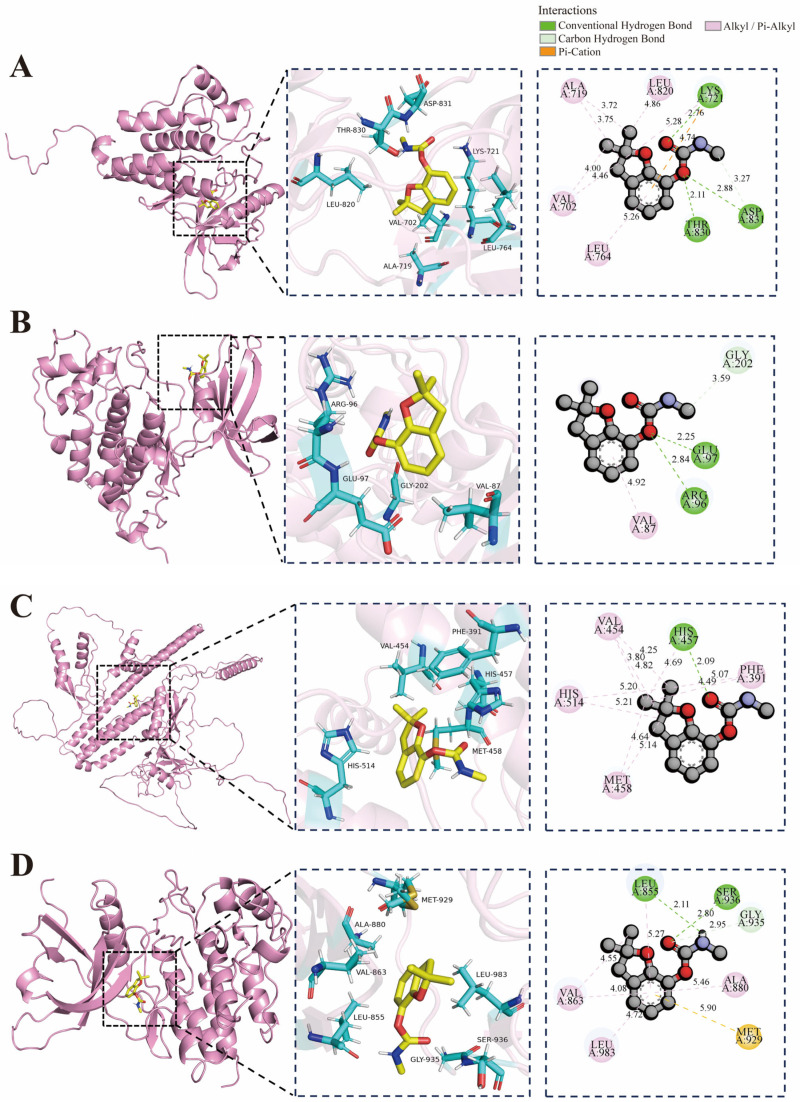
Molecular docking models. (**A**) Carbofuran with EGFR; (**B**) Carbofuran with GSK3B; (**C**) Carbofuran with APP; (**D**) Carbofuran with JAK2.

Molecular docking was performed for the top four core targets by degree score (EGFR: 1M14, GSK3B: 1J1B, APP: P05067 and JAK2: 3UGC) (Figure 3). Binding affinity was assessed by the calculated binding free energy, with more negative values indicating stronger interaction between the ligand and receptor. The binding energies of carbofuran with EGFR, GSK3B, APP, and JAK2 were −6.2, −6.1, −6.7, and −6.6 kcal/mol, respectively. All four docking runs yielded binding energies lower than −6.0 kcal/mol, indicating stable binding capability of carbofuran with the four target proteins and indicating potential high affinity of carbofuran for each identified target. Conformational analysis indicated that the carbofuran molecule stably embeds into the active pockets of each target protein. Carbofuran primarily binds to EGFR through hydrogen bonds formed with LYS721, ASP831, and THR830. Stable binding to GSK3B relies on hydrogen bonds between carbofuran and GLU97, ARG96, and GLY202. Interaction with APP is stabilized by alkyl bonds formed with VAL454, HIS514, MET458, and PHE391, as well as a hydrogen bond with HIS457. Carbofuran stably binds to JAK2 via hydrogen bonds with LEU855, SER936, and GLY935, supplemented by a sulfur bond with MET. However, these results remain computational predictions. Their biological significance and relative potency compared to known high-affinity inhibitors require further validation through in vitro binding or functional assays.

### 2.3. Untargeted Metabolomics

#### 2.3.1. Multivariate Statistical Analysis

**Figure 4:**
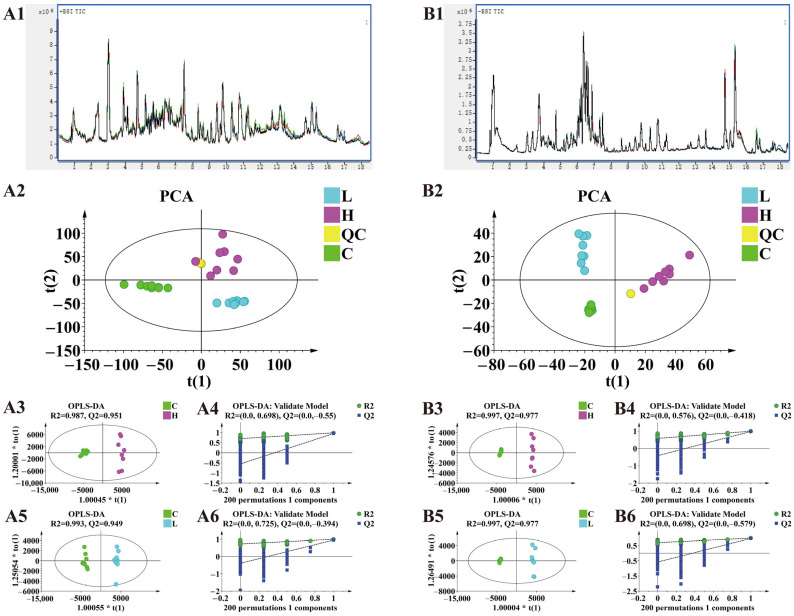
Metabolomics results. Panels A and B display the analytical results obtained in the positive and negative ion modes, respectively. (**A1**,**B1**) Total ion chromatograms (TIC) of the QC samples. (**A2**,**B2**) PCA score plots of all samples. (**A3**,**A4**,**B3**,**B4**) Orthogonal projections to latent structures-discriminant analysis (OPLS-DA) score plots for the high-dose group versus the control group and their corresponding permutation tests. (**A5**,**A6**,**B5**,**B6**) OPLS-DA score plots for the low-dose group versus the control group and their corresponding permutation tests.

The total ion chromatograms of all quality control (QC) samples during sample injection were examined, showing negligible drift in retention time and response intensity, indicating good instrument stability (Figure 4). Principal Component Analysis (PCA) was performed on the samples to reflect the original data state (Figure 4). Samples within each group showed good clustering, and there was a clear trend of separation between groups. The results indicated good sample reproducibility and significant differences between the different groups.

To further analyze the metabolic differences between groups and identify differential metabolites, OPLS-DA was employed. In both ESI+ and ESI- modes, clear separation was observed between the high-dose and control groups, and between the low-dose and control groups, confirming that carbofuran significantly affected mouse metabolism. R^2^ and Q^2^ indicated that the established models could explain the data matrix information and possessed good predictive ability. The OPLS-DA model was validated by 200 permutation tests. The results shown in Figure 4 indicate that the OPLS-DA model was meaningful, and the variable importance for the projection (VIP) values obtained based on this model were reliable and suitable for further analysis.

#### 2.3.2. Screening and Identification of Differential Metabolites

Differential metabolites were screened based on the VIP value from the OPLS-DA model and Permutation test, using the criteria VIP > 1 and *p* < 0.05. Differential metabolites were identified by matching the accurate mass and MS/MS fragmentation patterns with the Human Metabolome Database (HMDB) (Table 1). Compared to the C group, the H group identified 8 differential metabolites in positive ion mode and 4 in negative ion mode. The L group identified 6 differential metabolites in positive ion mode and 6 in negative ion mode. Four differential compounds were common to both the H and L dose groups: Phenylalanine, Niacinamide, Ornithine, and L-Tryptophan.

#### 2.3.3. KEGG Pathway Analysis of Differential Metabolites

**Figure 5:**
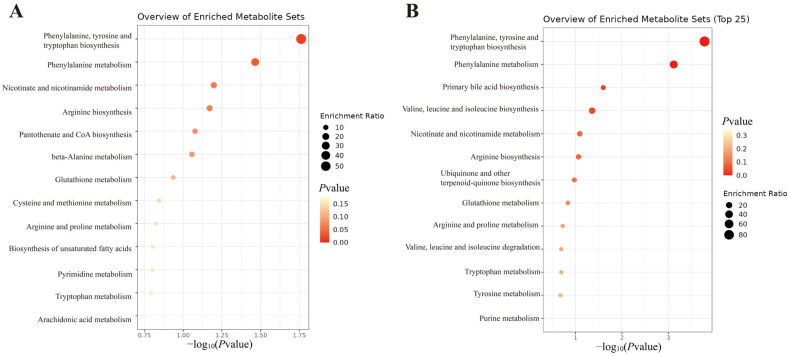
KEGG pathway enrichment analysis of metabolomics data. (**A**) Enriched metabolic pathways for the differential metabolites between the high-dose group and the control group. (**B**) Enriched metabolic pathways for the differential metabolites between the low-dose group and the control group.

To elucidate the biological relevance of the differential metabolites, KEGG enrichment analysis was performed using MetaboAnalyst 6.0 (Figure 5). The H group yielded 13 pathways, including Phenylalanine, tyrosine and tryptophan biosynthesis; Phenylalanine metabolism; Nicotinate and nicotinamide metabolism; Arginine biosynthesis; Pantothenate and CoA biosynthesis, etc. The L group yielded 13 pathways, including Phenylalanine, tyrosine and tryptophan biosynthesis; Phenylalanine metabolism; Primary bile acid biosynthesis; Valine, leucine and isoleucine biosynthesis, etc.

## 3. Discussion

Integrating the results of network toxicology and metabolomics, we found that carbofuran may induce ovarian toxicity through hormone disruption, oxidative stress, inflammatory response, and metabolic disturbance.

Endocrine disruptors can interfere with hormone synthesis, secretion, and feedback regulation, affecting the function of the reproductive endocrine system [25]. In the female reproductive system, the precise regulation of the hypothalamic–pituitary–ovarian (HPO) axis is crucial for maintaining normal menstrual cycles and fertility [26]. In this study, ELISA results showed a significant decrease in serum E2 levels along with elevated FSH and LH levels, indicating impaired feedback regulation of the HPO axis. Metabolomic analysis provided a potential metabolic explanation for these observations: significant disturbances were detected in pathways related to phenylalanine, tyrosine and tryptophan biosynthesis, phenylalanine metabolism, tyrosine metabolism, and tryptophan metabolism, manifested as upregulated levels of phenylalanine and tyrosine and downregulated tryptophan. These aromatic amino acids are key precursors for the synthesis of catecholamine (dopamine, norepinephrine) and indoleamine (serotonin) neurotransmitters, which precisely regulate the pulsatile release of GnRH from the hypothalamus [27,28,29,30]. We speculate that the imbalance in aromatic amino acid metabolism induced by carbofuran exposure may disrupt the homeostasis of these neurotransmitters, potentially affecting the secretory rhythm of GnRH, which could be one of the reasons behind the abnormal secretion of pituitary gonadotropins (FSH and LH). Concurrently, disturbances in the arginine and proline metabolism pathway suggest possible effects on nitric oxide (NO) synthesis, which plays a complex role in regulating ovarian blood flow and steroidogenesis [31,32]. Furthermore, NO may be involved in regulating E2 synthesis by inhibiting GnRH secretion and aromatase activity in ovarian granulosa cells [33]. Additionally, network toxicology predictions and molecular docking results indicate that carbofuran has a high binding affinity for the EGFR target. EGFR is a core component of the ErbB signaling pathway and is crucial for follicular development and granulosa cell function [34,35]. This suggests that carbofuran may also have the potential to directly interfere with local ovarian EGFR signaling, thereby affecting follicular development and estrogen synthesis. In summary, combined with histopathological observations of ovarian atrophy, reduced follicle numbers, and abnormal corpora lutea, this study indicates that the ovarian toxicity of carbofuran may stem from a multi-mechanistic network. Carbofuran may indirectly disrupt neuroendocrine regulation upstream of the HPO axis by disturbing aromatic amino acid metabolism; impair the ovarian microenvironment by affecting arginine and proline metabolism; and potentially directly act on ovarian targets such as EGFR, interfering with local signaling. Together, these pathways contribute to endocrine disruption, impaired follicular development, and structural damage to the ovaries.

Oxidative stress, resulting from an imbalance between ROS generation and clearance, can induce cell damage, lipid peroxidation, and DNA damage [36]. In the reproductive system, oxidative stress is a significant factor inducing follicular atresia, granulosa cell apoptosis, and inhibition of steroid hormone synthesis [37]. In this study, the enrichment of pathways such as chemical carcinogenesis—reactive oxygen species and lipid and atherosclerosis in network toxicology suggested the involvement of oxidative stress; disturbances in glutathione metabolism, arachidonic acid metabolism, and cysteine and methionine metabolism in metabolomics directly affect antioxidant defense and redox balance. Glutathione metabolism is a crucial intracellular antioxidant system, and its dysregulation may reduce ROS scavenging capacity, leading to ovarian oxidative damage [38,39]. Molecular docking revealed that carbofuran exhibits strong binding affinity for GSK3B. Given that GSK3B is a key regulatory node in the Nrf2/ARE pathway, a critical antioxidant response mechanism [40,41], our findings suggest that carbofuran may exacerbate ovarian oxidative damage by inhibiting GSK3B activity and consequently disrupting the Nrf2-mediated antioxidant defense system. In ovarian tissue, normal ROS levels play a key role in regulating follicular growth, angiogenesis, and steroid hormone synthesis [42]. An imbalance in ROS may affect the activity of ovarian steroidogenic enzymes. These results suggest that carbofuran may impair reproductive function by inducing ovarian oxidative stress.

The inflammatory response is the body’s defensive reaction to harmful stimuli, but chronic inflammation can lead to tissue damage, cell apoptosis, and hormonal disruption [43]. For the reproductive system, inflammatory cytokines can disrupt the ovarian microenvironment, impair follicular development, and even lead to ovarian failure [44]. In this study, enriched network toxicology pathways such as VEGF signaling pathway, fluid shear stress and atherosclerosis, and human cytomegalovirus infection indicated the involvement of inflammatory processes. Among these, the VEGF pathway is associated with promoting angiogenesis and inflammatory cell infiltration, affecting ovarian angiogenesis, follicular growth, etc. [45,46]. Arachidonic acid metabolism in metabolomics is the synthesis pathway for inflammatory mediators like prostaglandins and leukotrienes, and its disturbance may exacerbate the inflammatory response [47]. Molecular docking results demonstrated strong binding affinity between the core target JAK2 and carbofuran. As a key regulator of the JAK-STAT inflammatory signaling pathway, aberrant binding of JAK2 by exogenous compounds could lead to sustained pathway activation, promoting the release of pro-inflammatory cytokines and thereby contributing to local chronic inflammation in the ovary [48]. Furthermore, chronic inflammation can affect the release of pituitary gonadotropins and the ovarian response to these hormones, consistent with the elevated LH levels observed in this study [49]. The observation of ovarian interstitial hemorrhage and progressive structural damage in the high-dose group in histopathology provides morphological evidence for this local inflammatory response and the resulting tissue damage. This evidence indicates that carbofuran may activate inflammatory pathways, inducing local chronic inflammation in the ovary and exacerbating reproductive toxicity.

Metabolomics identified disturbances in multiple metabolic pathways, including Phenylalanine, tyrosine and tryptophan biosynthesis, Arginine and proline metabolism, biosynthesis of unsaturated fatty acids, and purine metabolism, involving amino acid, lipid, and energy metabolism. Disruption of phenylalanine metabolism affects the synthesis of neurotransmitters and hormone precursors; arginine and proline metabolism relates to NO synthesis and antioxidation; and the biosynthesis of unsaturated fatty acids affects cell membrane integrity and steroid hormone generation [31,50,51]. These extensive metabolic disturbances may lead to impaired cellular energy supply and anabolic disorders, consequently hindering follicular development and abnormal ovarian structure. The results suggest that carbofuran may induce reproductive toxicity by interfering with amino acid and lipid metabolism, disrupting ovarian energy and synthetic homeostasis.

In summary, this study integrated network toxicology and metabolomics to systematically reveal that chronic carbofuran exposure may induce ovarian toxicity through multiple mechanisms. However, our study has several limitations: metabolite identification relies on databases, potentially leading to false positives; the predictive results of network toxicology require further validation through in vivo and in vitro experiments. Subsequent research will focus on these issues to deeply explore the molecular mechanisms of carbofuran-induced ovarian toxicity.

## 4. Materials and Methods

### 4.1. Reagents

Carbofuran was purchased from Weiyel Inc (Henan, China). LC-MS-grade methanol, acetonitrile, and formic acid were purchased from Sigma-Aldrich (St. Louis, MO, USA). Ultrapure water was provided by Guangzhou Watsons Food & Beverage Co., Ltd. (Guangzhou, China). Mouse E2 and P4 ELISA kits were purchased from Wuhan Elabscience Biotechnology Co., Ltd. (Wuhan, China). Mouse FSH and LH ELISA kits were purchased from Shanghai Jianglai Biological Technology Co., Ltd. (Shanghai, China).

### 4.2. Animal Experiment

Female 6-week-old C57BL/6J mice were purchased from Chengdu Dashuo Experimental Animal Co., Ltd. All mice were housed under specific pathogen-free (SPF) conditions at a temperature of 22 ± 2 °C, relative humidity of 50 ± 10%, with a 12/12 h light/dark cycle, and were provided ad libitum access to food and water in the animal facility of West China Second University Hospital, Sichuan University. Furthermore, all experimental procedures were approved by the Experimental Animal Management and Ethics Committee of West China Second University Hospital, Sichuan University (Approval No.: 2022-043; Approval Date: 28 November 2022) and were conducted in accordance with the approved protocols and institutional animal welfare guidelines.

After 7 days of acclimatization, mice were randomly assigned to three groups (*n* = 8/group): Control group, Low-dose group, and High-dose group. Prior to administration, the carbofuran stock was dissolved in a small volume of dimethyl sulfoxide with the aid of sonication, and then diluted to the desired concentrations with normal saline (0.9% sodium chloride solution). The solution was vortexed thoroughly to ensure a homogeneous suspension. To maintain suspension stability, fresh carbofuran suspensions were prepared immediately before each gavage administration. Control group mice were gavaged with saline (0.9% sodium chloride solution), the Low-dose group with 0.5 mg/kg carbofuran, and the High-dose group with 2.0 mg/kg carbofuran, for 45 consecutive days, once daily. All mice were euthanized within 24 h after the final administration.

### 4.3. Sample Collection

Mice were anesthetized using isoflurane, and blood was collected via the orbital sinus. After standing at room temperature for 1 h, blood was centrifuged at 12,000 rpm to obtain serum. One portion of the serum was used for ELISA detection, and the remainder was stored at −80 °C for metabolomics analysis. Ovarian tissues were collected and fixed in formaldehyde for HE staining.

### 4.4. Network Toxicology Analysis

#### 4.4.1. Collection of Potential Compound Targets

Using “carbofuran” as the keyword, the 2D structure, SMILES string, and InChIKey were retrieved from PubChem (https://pubchem.ncbi.nlm.nih.gov/, accessed on 23 November 2025). Potential human targets were predicted using the comparative toxicology databases TargetNet (http://targetnet.scbdd.com/, accessed on 23 November 2025), Similarity Ensemble Approach (SEA) (https://sea.bkslab.org/, accessed on 23 November 2025), and SwissTargetPrediction (https://www.swisstargetprediction.ch/, accessed on 23 November 2025), with the species limited to “*Homo sapiens*”. Potential targets of carbofuran were predicted using the following databases under the specified conditions: TargetNet was employed with an AUC threshold ≥ 0.7 and the fingerprint type set to ECFP4 fingerprints. The SEA and SwissTargetPrediction databases were run with their default settings. The prediction results from all three databases were combined, and duplicate entries were removed to obtain the final set of potential carbofuran-related targets.

#### 4.4.2. Acquisition of Toxicity-Related Targets

Targets related to the keywords “reproductive toxicity”, “ovarian toxicity”, and “ovarian injury” were obtained from OMIM (http://www.omim.org/, accessed on 23 November 2025) and GeneCards (https://www.genecards.org/, accessed on 23 November 2025). Genes associated with the above-mentioned keywords were retrieved using the OMIM and GeneCards databases. The search results were then merged and duplicate entries were removed, yielding disease targets related to reproductive and ovarian toxicity. Drug- and disease-related targets were intersected using WeiShengXin (https://www.bioinformatics.com.cn/, accessed on 23 November 2025) to generate common targets, which were visualized in a Venn diagram.

#### 4.4.3. Protein–Protein Interaction Network Construction and Core Target Selection

Common targets were uploaded to STRING (http://string-db.org/, accessed on 23 November 2025), with the species set to “Homo sapiens” and a confidence threshold of 0.4 to generate the PPI network. The resulting data were imported into Cytoscape 3.10.4 software for visualization. Larger nodes with darker colors represent higher degree scores.

#### 4.4.4. GO and KEGG Enrichment Analysis

Common targets were subjected to GO and KEGG enrichment analysis using DAVID (https://davidbioinformatics.nih.gov/, accessed on 29 November 2025), with the species set to “*Homo sapiens*”. GO terms were sorted by −log(P) value, and the top ten biological processes were plotted as a bar graph. Similarly, KEGG pathways were sorted by −log(P) value, and the top 20 pathways were visualized as a bar graph.

#### 4.4.5. Molecular Docking

The molecular structure of the main active component was downloaded from the PubChem (https://pubchem.ncbi.nlm.nih.gov/, accessed on 29 November 2025) database and saved in SDF format. The 3D crystal structures of the target proteins were downloaded from the PDB database (http://www.rcsb.org/, accessed on 29 November 2025) and saved in PDB format. PyMOL 2.6.0 software was used to remove water molecules and ligands from the proteins, saving them in PDB format, and the Getbox Plugin was used to obtain docking pocket parameters. The processed protein and active component files were imported into AutoDock Tools 1.5.6, converted to PDBQT format. AutoDock Vina 1.1.2 was then used to perform molecular docking. Finally, PyMOL 2.6.0 software was used to visualize the molecular docking results.

### 4.5. Metabolomics Analysis

#### 4.5.1. Metabolomics Sample Preparation

A 100 μL aliquot of serum sample was transferred to a 1.5 mL centrifuge tube, followed by the addition of 300 μL methanol–acetonitrile (2:1, *v*/*v*) to precipitate proteins. After vortexing and centrifugation, 200 μL of the supernatant was transferred to an injection vial. An equal volume of supernatant from each sample was pooled to generate QC samples, which were analyzed alongside the study samples throughout the batch.

#### 4.5.2. HPLC-Q-TOF Analysis Conditions

We used an HPLC-Q-TOF system for untargeted metabolomics analysis. The HPLC-Q-TOF system consisted of an Agilent 1260 Infinity II high-performance liquid chromatograph coupled with an Agilent 6546 quadrupole time-of-flight mass spectrometer (Agilent, Santa Clara, CA, USA).

The column was a BEH C18 column (2.1 × 100 mm, 1.7 μm, Waters, Milford, MA, USA). Mobile phase A: water containing 0.1% formic acid, Mobile phase B: acetonitrile containing 0.1% formic acid, Flow rate: 0.3 mL/min, Injection volume: 5 μL. Gradient elution program: 2% B (0 min), 25% B (3 min), 30% B (5 min), 60% B (6–8 min), 90% B (15–18 min), and 2% B (18.5 min). Post-run time: 2 min. Electrospray ionization source (ESI). Detection was performed once each in positive and negative ion modes. Primary scan mass range: 50–1000 *m*/*z*, Speed: 1.40 spectra/s, Secondary scan normalized collision energy: 35 V, Mass window: 4 *m*/*z*, Speed: 4.00 spectra/s. Drying gas temperature: 200 °C, Flow rate: 14 L/min, Sheath gas temperature: 350 °C, Flow rate: 11 L/min, Nebulizer pressure: 35 psi.

#### 4.5.3. Metabolomics Data Analysis

Raw data were converted to ABF format using Analysis Base File Converter software (v.1.3.8802.34458). Subsequently, MS-DIAL software (ver.4.9.221218) was used for peak detection, peak extraction, retention time alignment, peak annotation, and signal drift correction of the plasma mass spectrometry data. Processing of the raw data yielded a dataset containing information such as molecular name, molecular weight, retention time, and relative abundance (peak area). Metabolite identification was based on matching exact mass and MS/MS spectra, corresponding to confidence level 2 of the Metabolomics Standards Initiative. The MS-DIAL software parameters were set as follows: the MS/MS spectral similarity score threshold was 80%, and common adduct ions such as [M + H]^+^ and [M − H]^−^ were considered. SIMCA software (ver.14.1) was used to perform PCA and OPLS-DA on the serum samples from each group. Screening criteria: VIP > 1 and *p* < 0.05 were considered differential metabolites. Fold changes (FCs) relative to the control group were used to evaluate metabolite regulation. An FC > 1.2 and an FC < 0.8 were set as the thresholds for defining up-regulation and down-regulation, respectively. Then, pathway enrichment of metabolic pathways was performed using MetaboAnalyst 6.0 (https://www.metaboanalyst.ca/, accessed on 27 November 2025).

### 4.6. ELISA Analysis

Levels of E2, P4, FSH, and LH in serum from mice were measured according to the experimental procedures described in the ELISA kit instructions.

### 4.7. HE Staining

Ovarian tissues were dehydrated through a graded series of ethanol solutions. They were then embedded and fixed in liquid paraffin, and sectioned to 4 μm thickness using a paraffin microtome. Subsequently, sections were deparaffinized in xylene and rehydrated through a graded series of ethanol solutions, followed by hematoxylin and eosin (HE) staining. Finally, sections were dehydrated through an ethanol gradient, sealed with neutral resin, and HE-stained ovarian tissue sections were obtained.

### 4.8. Statistical Analysis

All analyses were performed using SPSS 22.0 software. Results are presented as mean ± standard deviation (SD). Differences between groups were assessed using one-way analysis of variance (ANOVA). Statistical significance was set at *p* < 0.05. The normality of the distribution was confirmed using the Shapiro–Wilk test, and homogeneity of variances was verified by Levene’s test.

## 5. Conclusions

This study combined network toxicology and metabolomics approaches to investigate the toxic effects and mechanisms of chronic carbofuran exposure on the ovaries of female mice. The results showed that carbofuran caused ovarian histopathological damage, hormonal level disturbances, and altered metabolic profiles. Its toxic mechanisms involved multiple pathways, including endocrine disruption, oxidative stress, inflammatory response, and metabolic disturbance. Core targets such as EGFR, GSK3B, APP, and JAK2 may play key roles in the toxic process. This study provides new experimental evidence for a deeper understanding of the ovarian toxicity of carbofuran and offers a theoretical reference for the assessment of related health risks and the development of intervention strategies.

## Figures and Tables

**Table 1:** Identification results of potential differential metabolites in mouse serum under positive and negative ion mode.

No.	Rt (Min)	*m*/*z*	HMDB ID	Metabolites	Ion Mode	High Dose	Low Dose	*p*-Value
1	2.255	100.0756	HMDB0002039	2-Pyrrolidinone	POS	-	↓	9.62 × 10^−7^
2	6.032	115.05284	HMDB0000076	Dihydrouracil	POS	↑	-	1.45 × 10^−8^
3	1.072	118.08535	HMDB0003355	5-Aminopentanoic acid	POS	↓	-	8.45 × 10^−4^
4	1.016	123.06026	HMDB0001406	Niacinamide	POS	↑	↑	2.01 × 10^−7^
5	1.893	130.0891	HMDB0000687	Leucine	NEG	-	↑	1.64 × 10^−4^
6	6.207	133.11107	HMDB0000214	Ornithine	POS	↑	↑	2.46 × 10^−13^
7	3.252	146.05827	HMDB0029737	Indole-3-carboxaldehyde	POS	↑	-	9.63 × 10^−5^
8	2.327	166.08496	HMDB0000159	Phenylalanine	POS	↑	↑	2.13 × 10^−3^
9	1.035	167.0236	HMDB0000289	Uric acid	NEG	-	↑	1.49 × 10^−2^
10	2.942	181.05284	HMDB0000755	Hydroxyphenyllactic acid	NEG	↑	-	4.01 × 10^−4^
11	14.513	181.0681	HMDB0001889	Theophylline	POS	-	↑	3.17 × 10^−5^
12	1.45	182.0814	HMDB0000158	L-Tyrosine	POS	-	↑	3.98 × 10^−3^
13	2.942	203.08513	HMDB0000929	L-Tryptophan	NEG	↓	↓	4.00 × 10^−5^
14	3.408	295.12463	HMDB0000594	gamma-Glutamylphenylalanine	POS	↑	-	1.43 × 10^−3^
15	2.877	298.09293	HMDB0001173	5′-Methylthioadenosine	POS	↑	-	1.09 × 10^−5^
16	15.007	303.23489	HMDB0001043	Arachidonic acid	NEG	↑	-	5.34 × 10^−3^
17	8.619	391.29019	HMDB0000626	Deoxycholic acid	NEG	↓	-	1.59 × 10^−2^
18	7.255	407.2849	HMDB0000619	Cholic acid	NEG	-	↓	2.05 × 10^−2^
19	6.727	464.3075	HMDB0000138	Glycocholic acid	NEG	-	↑	3.52 × 10^−3^
20	6.727	498.2955	HMDB0000951	Taurochenodesoxycholic acid	NEG	-	↑	3.14 × 10^−2^

Arrows indicate the trend of metabolite change in the corresponding dose group compared with the control group (↑: up-regulated, ↓: down-regulated, -: indicates that the metabolite did not meet the differential screening criteria in that dose group).

## Data Availability

The raw data supporting the conclusions of this article will be made available by the authors on request.

## Abbreviations

The following abbreviations are used in this manuscript:

E2	Estradiol
P4	Progesterone
FSH	Follicle-Stimulating Hormone
LH	Luteinizing Hormone
ELISA	Enzyme-Linked Immunosorbent Assay
HPO	Hypothalamic–Pituitary–Ovarian
LC-MS	Liquid Chromatography–Mass Spectrometry
PPI	Protein–Protein Interaction
GO	Gene Ontology
KEGG	Kyoto Encyclopedia of Genes and Genomes
EGFR	Epidermal Growth Factor Receptor
GSK3B	Glycogen Synthase Kinase 3 Beta
APP	Amyloid Beta Precursor Protein
JAK2	Janus Kinase 2
PCA	Principal Component Analysis
OPLS-DA	Orthogonal Projections to Latent Structures–Discriminant Analysis
VIP	Variable Importance for the Projection
HMDB	Human Metabolome Database
QC	Quality Control
ESI	Electrospray Ionization
HE	Hematoxylin and Eosin
SD	Standard Deviation
ANOVA	Analysis of Variance
ROS	Reactive Oxygen Species
Nrf2	Nuclear Factor Erythroid 2–Related Factor 2
ARE	Antioxidant Response Element
